# Genome Sequence of *Borrelia parkeri*, an Agent of Enzootic Relapsing Fever in Western North America

**DOI:** 10.1128/genomeA.00018-14

**Published:** 2014-02-13

**Authors:** Alan G. Barbour, Shelley Campeau Miller

**Affiliations:** Departments of Microbiology & Molecular Genetics and Medicine, University of California, Irvine, Irvine, California, USA

## Abstract

*Borrelia parkeri* is a relapsing fever agent that rarely causes human infection, unlike other North American species. *B. parkeri* strain HR1 was isolated from *Ornithodoros parkeri* ticks. The sequences of its linear chromosome and large plasmid were determined by next-generation sequencing. These confirmed its closer relatedness to *Borrelia turicatae* than to *Borrelia hermsii*.

## GENOME ANNOUNCEMENT

Several species of the spirochete genus *Borrelia* cause relapsing fever (RF) in humans, domestic animals, and wildlife in North America and on other continents (1). *B. parkeri* has infected horses and its natural rodent reservoirs (2, 3), but reports of human infection are rare (4). The sequences of its 16S rRNA suggest a close relatedness of *B. parkeri* to the more commonly reported agent *Borrelia turicatae* (4, 5). Besides ecological differences in their distributions in North America, the two species differ in their biological features, such as their propensity for transovarial transmission in their tick vectors (6). RF *Borrelia* species have linear chromosomes of ~1 Mb and large linear plasmids of >120 kb (1).

*B. parkeri* strain HR1 was recovered from *Ornithodoros parkeri* ticks collected from *Spermophilus beecheyi* burrows in the Carmel Valley of California. A pooled homogenate from the ticks was injected into severe combined immunodeficient mice (C.BKa-*Igh*^*b*^/IcrCrl). A blood isolate was subsequently cloned by a limiting dilution and then cultivated in axenic medium, as described previously (7). Genomic DNA was isolated using a Qiagen DNeasy kit (Valencia, CA). Two libraries were generated separately and sequenced on Illumina (Hayward, CA) instruments, the Genome Analyzer IIx at Ambry Genetics (Aliso Viejo, CA) and the Illumina HiSeq 2000 at the University of California, Irvine, as described previously (8, 9). The outputs were combined for a total of 87,630,260 paired-end reads of ~100 nucleotides (nt), with an estimated coverage of ~600×. Using the Assembly Cell algorithm of CLC Genomics Workbench version 6 (CLC bio, Denmark), these were assembled *de novo* into 5 contigs totaling 907,128 bp, as well as 3 contigs totaling 108,882 bp for the linear plasmid. Short gaps between the contigs were filled in by mapping reads to the *B. turicatae* chromosome (GenBank accession no. CP000049) or to its large plasmid (accession no. HM008710). The sequence redundancies between the *de novo* and mapped-to-reference assemblies were identical. For the chromosome, annotation was performed by the Prokaryotic Genome Annotation Pipeline (http://www.ncbi.nlm.nih.gov/genome/annotation_prok), followed by manual annotation. For the plasmid, the prediction of protein-coding sequences (CDS) was performed by MetaGeneAnnotator (10), followed by manual annotation.

The linear chromosome sequence comprises 916,945 bp, with a G+C content of 28.9%. The chromosome contains 825 CDS, 32 tRNAs, and 3 rRNAs, which are syntenic with those of the chromosome of *B. turicatae*. A GC skew shift at ~459 kb was consistent with an origin of replication. The pairwise DNA distances determined by DNADIST (http://evolution.genetics.washington.edu/phylip) for the *B. parkeri* chromosome at 911,986 aligned sites were 0.022 from the chromosome of *B. turicatae* and 0.091 from the chromosome of *Borrelia hermsii* (accession no. CP000048). The 113,739 bp constituting the leftmost three-quarters of linear plasmid lp150 had CDS for plasmid partition proteins, including ParA, which were orthologous to those of large plasmids of other RF *Borrelia* species (9). Chromosome and plasmid comparisons established *B. parkeri* as a sister species to *B. turicatae*.

### Nucleotide sequence accession numbers.

The complete chromosome sequence of *B. parkeri* HR1 and the sequence of 113 kb of its lp150 plasmid have been deposited in the GenBank/DDBJ/EMBL database under accession no. CP007022 and CP007036, respectively, as part of BioProject PRJNA231102.
